# “I am used to being extremely patient because I’m forced to be”: the affective politics of accommodation for disabled archivists

**DOI:** 10.3389/fsoc.2025.1468401

**Published:** 2025-06-13

**Authors:** Julia Rose Karpicz, Tara Brar, Gracen Mikus Brilmyer, Veronica L. Denison

**Affiliations:** ^1^Department of Undergraduate Education Initiatives, University of California, Los Angeles, Los Angeles, CA, United States; ^2^Disability Archives Lab, School of Information Studies, McGill University, Montreal, QC, Canada; ^3^Rhode Island College, Providence, RI, United States

**Keywords:** disability, archival studies, critical access studies, emotional labor, archival access, workplace accommodation, disability accommodation

## Abstract

Archives—repositories that store, organize, and give access to historical materials—produce a constellation of affects for both the people who use them and work within them. This article, drawing on data collected through semi-structured interviews with 12 disabled archivists in Canada and the United States, focuses on how disabled archival workers experience, manage, and perform emotions while navigating work-related access and accommodation in archival institutions. The ineffectiveness of traditional systems of individual accommodation—which sometimes forced them to disclose their access needs or, alternatively, feel pressured into denying their own needs—produced complex emotional responses among participants. Many spoke about the emotional toll of requesting accommodations, while others described their exhaustion and refusal to engage with such processes. Yet, participants highlighted how collective (rather than individual) approaches to access transformed the affective experience of access towards ease and empowerment. Centering this affective reality for many disabled archivists, this research echoes the growing body of research and theory around access labor, while also adding focus on *the affective debt of archival access* that occurs through accommodations processes—both an internal indebtedness, where one “borrows against” their patience and energy to survive, and an external indebtedness, where one is required to “pay” in gratitude, vulnerability, and being nice in order to be deserving of accommodation. We draw attention to how the very people who facilitate access to historical documents are also navigating their own access—performing additional forms of labor to manage inaccessible, precarious, or hostile work while also imagining access otherwise.

## 1 Introduction

In her book *Crip Spacetime*, Margaret Price describes how “emotionally devastating” it is for disabled employees in higher education to experience “the nearly constant dissonance of being assured that accommodation is a straightforward, legally protected process while also navigating the endless obstacles and sometimes open cruelty encountered along the way” (p. 120). Accommodations can be a wide range of formal modifications that are made to remove barriers and facilitate access for community members with a variety of disability experiences, including physical, mental, and developmental disabilities. In the United States and Canada, national and local legislation can require that places open to the general public, such as schools, hospitals, businesses, and workplaces, have a process for accommodating people with disabilities.^1^ Though meant to facilitate full and equal participation, accommodation processes—whether for work, education, or daily life—are often complicated, bureaucratic, difficult, and insufficient (Titchkosky, 2011). For example, in Price’s study, disabled academics highlighted a wide range of economic and noneconomic costs and harms they incurred while seeking accommodations at work. Primary among these were emotional costs. As Price explains, the “emotional costs for disabled employees are high in part because they must work so hard, and often in very personal and emotionally charged ways, to negotiate access” (p. 123). Echoing and building on this work, we focus on the emotional and/or affective, impacts of navigating access within archives—repositories that store, organize, and give access to historical materials. Archives produce a constellation of affects: from the ways marginalized communities feel erased through the ways they are underrepresented or misrepresented in archival materials (e.g., Caswell et al., 2016), to the ways disabled archival users feel the violences of the past—histories of institutionalization, medicalization, and spectacularization—as embodied through records (Brilmyer, 2021; Rinn, 2018), archives shape users’ sense of themselves, others, and history. Archival spaces, through their partial or complete inaccessibility, can produce feelings of alienation for disabled archival users (Brilmyer, 2022). Disabled archival workers, in particular, have long been integral to understanding disability in archival material, preserving disability histories, and building and facilitating accessibility in reading rooms.

This article focuses on archival workers: drawing on interview data as well as archival and disability studies scholarship to highlight how disabled archival workers experience accommodation processes and their affective responses to this landscape across different archival institutions. We first explore relevant ways of understanding labor, drawing from works in archival studies that illustrate the many affects of archives and the ways archival labor is understood in addition to works in disability studies’ that explore access labor, as in “the work and effort that goes into making things accessible” (Fink, 2020). After detailing the methods for this research—semi-structured interviews with disabled archival workers at a range of archival institutions in Canada and the United States—we then outline two main clusters of findings. First, we highlight the many ways that interviewees spoke about seeking accommodations, sometimes being forced to disclose their access needs or, alternatively, feeling pressured into concealing them and denying their own needs. Second, we illustrate archivists’ responses to their experiences with accommodations: the emotional toll, their refusal to and exhaustion with such processes, and the collective nature of access that is possible. Together, this research echoes much existing work on access labor, while also adding focus on *the affective debt of archival access* that occurs through accommodations processes—both an internal indebtedness, where one “borrows against” their patience and energy to survive, and an external indebtedness, where one is required to “pay” in gratitude, vulnerability, and being nice in order to be deserving of accommodation.

## 2 Literature and theoretical background

### 2.1 Archives, affect, labor

Archivists shape and are shaped by their work. While there is a growing body of literature that emphasizes the ways that archival users are impacted by archives, archivists have also drawn attention to the ways that archival work is multifaceted and involves many types of labor. In general, archivists perform a variety of tasks—from appraising, describing, processing, outreach, and helping users and giving access to materials, to name a few; archival labor takes many forms. In addition, recent scholarship has begun to address the affective—the internal, visceral, and/or emotional aspects that shape someone’s experience of the world, themselves, and relations to power (Pedwell and Seigworth, 2023) —impacts of archives. Marika Cifor, for one, implores the archival field to center affect:

“In order to be accountable to the individuals and communities that are affected, and to live up to the obligations of facilitating larger societal reckoning processes, the archival field needs to expand its ethical orientation to address considerations of emotional justice (Cifor, 2016, p. 9).”

While many have drawn attention to the affective impacts of archival users (e.g., Brilmyer, 2022; Gilliland, 2014; Guerrero, 2022; Caswell et al., 2016; Caswell et al., 2017; Cifor and Gilliland, 2016), we focus here on the affective dimensions of archival workers. We think about affect and emotion as referencing similar phenomena: internal experiences of emotion, intentional performance of emotions, how emotional experiences are shaped by power and through ableism, and how the emotions surrounding accommodation processes within archival institutions converge in a pattern. This understanding reflects an understanding of affect “as part of what emotions do” (Schmitz and Ahmed, 2014, p. 97), in the sense that the emotions that arise in response to another “do not respond the way they do because of the inherent characteristics of others: we do not respond with love or hate because others are loveable or hateful. It is through affective encounters that objects and others are perceived as having attributes, which ‘gives’ the subject an identity that is apart from others” (Ahmed, 2014, pp. 52–53).

The emotional landscape around workplace accommodation reflects a broader history of affective encounters around disability and access. For example, when employers treat access as charity and burden, this way of orienting is historically rooted and results in the negotiation of several emotions tied to charitable giving: feeling generous, feeling thankful, feeling patient, feeling humility. Within archival institutions, these histories of encounter are particularly salient because archival workers negotiate them through real-time emotional encounters while also encountering them through historical records about disability and disabled people (Brilmyer, 2021). We refer to this complex landscape as the affective politics of accommodation.

This study contributes to a growing body of scholarship and conversations around the underrecognized forms of labor that archivists regularly perform, such as navigating the emotional and traumatic elements of archival work (i.e., Arroyo-Ramírez et al., 2021; Caswell and Cifor, 2016; Guerrero, 2022). Trauma has come to the fore in the archival landscape to draw attention to the felt realities of archivists. For those processing violent histories, traumatic collections can cause distress (Regehr et al., 2023; Nathan et al., 2015). In their report on the international landscape of trauma and archives, Nicola Laurent and Kirsten Wright highlight how most archivists they surveyed have experienced “distressing content, distressing situations (for example, a distressing interaction with an archives user) and experiences of vicarious trauma” (Laurent and Wright, 2023). Others have highlighted how archivists experience secondary trauma both by processing traumatic materials as well as supporting or interacting with users, donors, or creators (Lassere and Whyte, 2021; Laurent and Hart, 2020; McCracken and Hogan, 2021). They state how participants reported feeling like they should “tough it out” or questioned whether some of their upsetting experiences “qualified as traumatic” (Sloan et al., 2019, p. 13). Importantly, archival workers in many of these studies have reported not being taught about trauma in their archival education^2^ or professional development, not receiving support at their organizations around traumatic materials and their emotional well-being, or even being “discouraged [from] talking about emotional matters on work hours.” (Sloan et al., 2019, p. 14).

In addition to exploring the emotional impact of navigating traumatic content within archives, scholars have also highlighted other forms of emotional labor that constitute a significant part of archivists’ roles (e.g., Douglas et al., 2019; Lowry, 2019). For example, the practices of empathy that archivists engage in as they form and navigate relationships with creators, donors, users, and communities have become an important area of focus within critical archival studies. In 2016, Michelle Caswell and Marika Cifor proposed “radical empathy” as a core tenet to archival work within “a web of affective responsibilities” for archivists. Their introduction of a feminist ethics of care has been widely taken up and was revisited in a 2021 issue of the *Journal of Critical Library and Information Studies*, where contributors illustrated, shifted, and expanded how this framework could be applied and envisioned. In the issue, contributors mark the many people, affects, and politics that archival work involves and the responsibilities that archivists have in stewarding materials for various communities (Arroyo-Ramírez et al., 2021).

Although this growing body of scholarship recognizes the complexity of archival labor, archival work has historically been undervalued and often invisibilized. Michelle Caswell, critiquing the ways that scholars in the humanities have routinely erased the labor of the archivists who support their research, highlights how, “almost none of the humanistic inquiry at ‘the archival turn’ (even that which addresses ‘actually existing archives’) has acknowledged the intellectual contribution of archival studies as a field of theory and praxis in its own right, nor is this humanities scholarship in conversation with ideas, debates, and lineages in archival studies.” (para 4). Tracing the lineage of how archivists have historically been deemed “handmaidens of history”—expected to “be an invisible caretaker, a docile handmaiden,” (Cooke et al., 2021, p. 507)—Lapp (2019) shows the ways in which nineteenth-century characterizations of archival neutrality invisibilized the work of archivists in support of the masculinized work of historians. She articulates how this is not a phenomenon of the past, that:

In the field of library and information studies, a rhetoric of cultural caretaking as the purview of white, educated, middle-class women continued well into the twentieth century constructing and perpetuating the “ideal archival worker” through reified categories of race, gender, sexuality, class, and ability.

Thus, many scholars and practitioners have been critical of the guise of neutrality behind archival work—that also makes possible the invisibilization of labor^3^—and have highlighted the ways archival norms exclude many ways of knowing, working, and being. In other words, as Elvia Arroyo-Ramírez, Jasmine Jones, Shannon O’Neill, and Holly Smith point out, “As practitioners in this field, we have inherited a professional and institutional culture of toxic ambition,” one that exploits, underpays, or expects free labor from students and early professionals, over-relies on contract work and low wages, and prioritizes “hyper-productive approaches over slow and deliberate work,” amongst many other things (Arroyo-Ramírez et al., 2021, p. 2–3). S. Williams pinpoints the slow ways in which such norms might change:

Perhaps we are so terrible at advocating for the importance of what we do because to be good at that advocacy means acknowledging that the manner in which we conduct this labor is often times unequal, rooted historically in sexism, racism, ableism, and classism, and that will always present a challenge to the access we hope to provide.

Yet, many are pushing back. Arroyo-Ramírez et al. powerfully note, “As an archival professional, you are meant to keep a straight face, a stiff upper lip, to toe the line. We reject this.” Instead of complying with and maintaining professional norms that undergird harmful practices and the status quo of the profession, archival scholars and practitioners are identifying the many harmful aspects of the archival profession, the impacts on archival workers in addition to users, and the ways that practice and the profession needs to change today and into the future.

### 2.2 Access labor and the actualities of accommodation

Just as archival scholars and practitioners are challenging constructions of archival work as neutral and objective, so too are critical disability studies and critical access studies scholars expanding beyond the construction of access as a neutral object, “a substance to be measured for its presence or absence, as exemplified by the ‘yes/no’ check box found on university website descriptions of classrooms” (Titchkosky, 2011, p. 41). While early scholarship and activism around access focused on advocacy and “making the case” for physical accessibility standards and universal design approaches (Hamraie, 2017), in recent years, scholars and activists have increasingly documented and theorized access as a process (e.g., Acton et al., 2021; Fink et al., 2020; Schalk, 2017). In this theorizing, the process of access is as much about practices and tools that create access as it is about the “politics of knowing” that shapes how access is understood, recognized, and facilitated within society (Hamraie, 2017, p. 14).

As part of this shift, scholars have explored the political and relational nature of access work, for example, documenting how accessibility guidelines and standards were originally developed to accommodate and facilitate the participation of white disabled veterans in public institutions (Williams, 2016); how traditional approaches to accommodation require an inordinate amount of administrative labor and are designed to be intentionally cumbersome, complex, and costly (Emens, 2021; Price, 2021; Titchkosky, 2011); how power dynamics with supervisors and the precarity of employment status shape decisions around disability disclosure (Damiani and Harbour, 2015); and how rights-based, individualized approaches to accommodation have reduced understandings of access to a set of procedural and logistical considerations (Mingus, 2012; Valentine, 2020). Through this research, scholars have developed several concepts to help describe access as a relational and political phenomenon. For example, Emens (2021) conceptualizes the labor involved in maintaining disability benefits as a specific category of what they call “life admin,” as in “all the office-type work that it takes to run a life…like scheduling and ordering and answering calls and filling out forms [as well as] long-range planning and financial decision-making” (p. 2335). Emens (2021) highlights “disability admin” as distinct because of the amount of labor that is demanded to not only manage access to formal services and accommodations but also, more broadly, to negotiate access in day-to-day life.

Importantly, as scholars document the experiences of disabled people with navigating access and accommodation, they have challenged the way disability legislation in the United States has framed accommodation as inherently benefitting disabled individuals, without considering the ways formal accommodations can be logistically, financially, and emotionally burdensome for individuals (Emens, 2021). For example, Price’s (2024) recent study about the experiences of disabled faculty with accommodation vividly captures how university accommodation processes are designed to be delayed, restrictive, and complex, despite being framed as linear and straightforward. Price (2024) explains how, because of this design, disabled faculty are forced to incur several types of costs, including additional administrative labor such as coordinating appointments to get documentation, financial costs related to self-accommodation, the relational injury of insulting and demeaning interactions with colleagues and administrators, as well as the stress and frustration of living through these processes. As Price (2021) explains, these harms put disabled faculty out of time with the normative timelines of the university, creating an experience of professional life that is “extraordinarily hard to understand from a nondisabled point of view” (p. 263). For several faculty members in Price’s study, the misalignment between processes of accommodations and the expectations of university culture forced disabled faculty out of the academy altogether.

#### 2.2.1 Access labor as emotional labor

In documenting the actualities of requesting accommodation, disability studies has pushed towards a more robust conceptualization of *access labor*, which Fink (2020) defines as “the work and effort that goes into making things accessible.” Significantly, scholars and activists have documented the political, emotional, and relational components of access labor that are enmeshed in what has traditionally been understood as a purely logistical and administrative process (Emens, 2021). This paper expands upon literature highlighting the emotional labor implicated in negotiating access. In doing so, we weave the study of access labor into broader ethnographic and qualitative research about workers’ experiences of emotional labor.

Wharton (2009) describes the sociology of emotional labor as fundamentally concerned with “understanding how emotions are regulated by culture and social structure and how emotional regulation affects individuals, groups, and organizations” (p. 148). Building on the foundational work of Hochschild (1983), research on emotional labor at work has historically focused on how workers in service industries are expected to manage their feelings as part of interacting with the public (Wharton, 2009). This literature offers several concepts that are relevant to the study of emotional labor within accommodation processes, most notably *surface acting*, wherein workers project an emotion different from what they feel to manage others’ feelings (Hochschild, 1983).

Several studies have sought to measure the “affective requirements of jobs… the degree to which workers’ perceive their jobs as requiring them to display certain emotions or be sensitive to the emotions of others” (Wharton, 2009, p. 158). This scholarship has mainly focused on emotional labor as it is performed in relationship to clients or customers—in roles such as service work (Leidner, 1999), care giving (Sass, 2000), and customer service (Totterdell and Holman, 2003). Scholars have detailed how these service-oriented industries have implicit and, sometimes, explicit expectations that workers will manage their own emotions, perform emotions that they may not feel, and manage or elicit customer or client emotions (Hochschild, 1983; Sass, 2000). Managing the emotions of others serves the interest of service-oriented workplaces by facilitating the compliance, comfort, dignity, and satisfaction of customers and clients (Leidner, 1999; Sass, 2000). Scholars have documented that when this type of emotional management masks conflicting internal feelings, meaning workers are required to perform emotions they do not feel, it often leads to burnout (e.g., Glomb and Tews, 2004; Ozcelik, 2013).

Yet, there has been limited research on how workplace structures require workers to engage in emotional labor with colleagues, rather than clients, and how this labor affects workers (Gabriel et al., 2020; Ozcelik, 2013). This study contributes to this emergent sub-area by considering how disabled workers engage in emotional labor to facilitate access to work itself. Put another way, the emotional labor that this study explores is distinct because (1) disabled archivists are engaging in emotional labor to remove barriers to doing their job, and (2) this labor is not a standard, work-related responsibility for all archivists. Thus, this study offers insight into how ableist norms create disparate experiences of emotional labor at work.

In addition to building with research on emotional labor in the workplace, this study extends emerging scholarship within disability studies on the emotional costs of accommodation for people with disabilities. Konrad (2021) documents “how a lack of familiarity with disability and practices of accessibility places pressure on disabled people to teach others how to participate in access” (p. 183). They note that “the specific labor of involving others in accessibility” requires four rhetorical techniques—a performance of self, confronting audience reactions, a value exchange, and rhetorical pedagogy—that all hinge on the moment-to-moment capacity of disabled folks to deflect and manage the harmful affective politics of dis/ableism (p. 183). For example, this labor might involve mitigating the anger and paternalism that arises in response to naming access barriers by performing a polite, calm, and knowledgeable disabled self (Konrad, 2021). In highlighting the fatigue that accumulates through this labor, Konrad (2021) makes the point that energy and emotional labor required to navigate social spaces often reflects the contours of power and oppression within those spaces.

By studying the energy and emotional labor that is demanded in traditional processes of accommodation—in addition to the emotional labor that archivists may perform as part of their work— we can trace and better understand how power and (in)equity function within organizations. By focusing on access labor within archival institutions, this study dialogues with existing research on accommodation processes for public benefits (Emens, 2021) and within university spaces (e.g., Titchkosky, 2011; Dolmage, 2017) by documenting how emotional management functions as part of the labor of access for disabled archival workers.

## 3 Materials and methods

Engaging and building on the aforementioned literature on affect, archives, labor, and accommodations, this article draws on data collected through semi-structured interviews with 12 disabled archival workers. Participants were recruited through archives-related listservs and social media. To qualify, participants had to be located in the US and Canada, and needed to (a) self-identify as disabled, (b) have worked as an archivist or an archivist-related job within an archive, special collection, or museum or completed an archives-related degree (such as a Masters of Archival Studies or a Masters of Library and Information Science) in the past 15 years in the US and/or Canada, and (c) be at least 21 years of age at the time of recruitment. Interviewees were paid $50 CAD for their time, could also specify access needs and if they wanted to be interviewed by either or both PIs and a student research assistant, and were given the interview questions in advance. Each interview was conducted using video conferencing software, lasted 60–90 min, and was recorded with the consent of each participant. The recordings were transcribed, and the transcripts were collaboratively coded by the research team using coded methods based in grounded theory such as open coding, axial coding, and focused coding (Saldana, 2015; Charmaz, 1994; Glaser and Strauss, 2009; Thornberg and Charmaz, 2013). Through our collaborative and iterative coding process, we located clusters of codes and discussed their definitions, differences, and relationships, as we made sense of the data; these major themes shaped this article and others (Brilmyer et al., 2024; Denison et al., 2024).

As we iteratively coded the transcripts we also reflected on our own experiences as disabled people researching disability. We recognize how our own positionalities inform how we interpret these conversations, that some of the situations we report on are also personal or familiar to some of us, yet, we also recognize how we are each coming with our own differing experiences, intersecting identities, and politics around how we understand disability, archives, and labor. Julia is a mixed-race, Black, disabled, and cisgender woman with a professional background in postsecondary disability services. Her scholarship uses qualitative methods to explore access labor within U.S. higher education institutions. Tara identifies as a disabled, neurodiverse, brown, and Canadian woman. Gracen is a white, non-binary, disabled, chronically ill, and neurodivergent person currently working in academia and from a middle-class background. They write from their position of organizing, researching, and building community in both archival and disability spaces. Veronica identifies as a white, disabled, cisgender woman who was a first-generation college student and works as an archivist in academia. Her research centers around access and use in the archives, as well as trauma-informed archival practices. Tara identifies as a disabled, neurodiverse, brown, and Canadian woman. As a team of disabled researchers with a wide range of experiences and intersecting identities, we are both insiders and outsiders to this research—we recognize how we share some experiences of access and accommodations with our participants, but also try to honor the differences that each of our experiences brings.

Importantly, as we navigate our interpretations of the interviewees’ words, we center a process of ongoing consent, where each participant has multiple opportunities to approve and edit their words and our analysis of them. Each interviewee read and signed a consent form before the interview, which we also went over together at the beginning of each conversation to answer questions or provide clarification. For each manuscript we write using their words, we first send them a copy of all the quotes we plan to use and then a copy of the full manuscript. During each, they can change how they want to be cited (by name, an alias, or anonymously), edit or remove any of their quotes, and suggest changes to the ways we interpret their words or each manuscript overall. Participant edits are prioritized in this piece as they clarified their ideas and further reflected on their experiences, and we take their feedback seriously. Our hope is that with multiple rounds of review, that the interviewees see their words reflected in ways that feel true to them as well as feel the collaborative nature of this research as it could not be done without their powerful reflections on their lived experiences.

## 4 Results

What lays a foundation for the findings that follow is how, in many of the interviews, archivists described their places of employment as professional environments built around a culture of compulsory abledness (McRuer, 2018). This is not to say that some workplaces were not accommodating or openly hostile, or that accommodations were necessarily unavailable, but that the general ethos assessed from the interviews emphasizes the common experience of inaccessible workplaces, laborious accommodations processes, and other ableist norms that participants experienced. While this culture was reflected in a variety of organizational and interpersonal norms, a defining aspect of this culture was the frequent lack of effective accommodation processes across departments. For example, several participants described it as common practice for archival institutions to assume that job applicants would not require accommodation. For one, Michelle Ganz, a mixed race (Indian and Polish) disabled woman who is severely deaf and very nearsighted and wears assistive devices, a hearing aid and glasses to interact with the world, explained how, out of hundreds of interviews, there was only one institution “where someone actually asked me if I’m [needing] any sort of accommodation. Everybody else just assumed I would tell them if I needed so, or figure it out as I went along” (Denison et al., 2024, p. 299). Another interviewee, Joy Rowe —a cisgender queer woman in Canada with hearing aids, described herself as a white settler who is unprecariously housed and employed, with all material and social needs met— similarly described how at the archives in which she worked: “There’s no formal process at all, but you really just need to ask for what you need. And there’s not a lot of–I mean, you cannot really ask directly, but eventually you’ll–some needs are met.” Once disabled archivists were hired, this ableist professional culture often extended into the workplace. Interviewees frequently recalled how their attempts to address access barriers and engage in an accommodation process resulted in a lengthy and onerous struggle in which their access needs often remained unmet. Participant 2, who has multiple invisible disabilities (psychiatric, neurodevelopmental, and musculoskeletal), an archivist with “an amalgamation of physical and mental disabilities” explained how even “just the simplest facilities fix was unbelievably time-consuming and lengthy and bureaucratic.”

With this background, the following two clusters of findings center the narratives of disabled archivists as they conveyed how resistant archival institutions were to facilitate access as well as the layers of access labor that were regularly exacted from disabled workers. The first finding highlights the lived experiences of disabled people navigating the accommodations process. Specifically, interviewees described being forced to disclose their access needs repeatedly and publicly and compelled to conceal their access needs or “power through” work without accommodation. The second finding illustrates archivists’ responses to their experiences with accommodations: the emotional toll, their refusal and exhaustion with such processes, and the collective nature of access that can be possible. Interviewees also conveyed how emotional labor was entangled in negotiating access at work, including processing internal feelings of hurt, anger, and stress and managing the emotions of others through patience and gratitude. While individual accommodation processes were a source of frustration and difficulty for all interviewees, several found a sense of purpose and confidence in shifting their efforts towards developing a culture of access at work and improving access for future disabled colleagues.

### 4.1 Navigating the accommodation process

#### 4.1.1 Forced to (repeatedly) disclose

Many formal accommodations processes involve a component of disclosure, the naming of an access need and justification of that need through personal narrative and frequently biomedical documentation. However, interviewees experiences of disclosure extended far beyond a confidential process with human resources; several described being forced to discuss their disability and access needs day-to-day at work as part of an ongoing process of negotiating access in their workplace. This repeated disclosure was often compelled because the department’s day-to-day work took for granted a certain set of abilities, e.g., the ability to process information verbally or to use steps to access different floors within the archives. As a result, although accommodation processes in the workplace are intended to be confidential, several participants described how often they were either forced to disclosure or had their disability information disclosed by others in front of coworkers.

These forced disclosures included, for one, supervisors and colleagues publicly asking questions about participants’ access needs. For example, Participant 3, a white woman, from a middle class background, who has an invisible disability (dyslexia), recalled a supervisor discussing their accommodations in front of another colleague without her consent:

Having someone that you disclosed to be like, ‘Oh, do you still want that [accommodation]?’ And I was like, ‘Oh, well, yes,’ but like, maybe this wasn’t the moment to talk about it? Maybe you should have asked me if I had talked to the other person before you brought it up in this scenario? That’s where I have a little bit more of a—that wasn’t very professional and that wasn’t the nicest move, you know?

Workplace accommodations are confidential processes, which means that only individuals involved in the administration of an accommodation, such as Participant 3’s supervisor, would be provided with information about an employee’s access needs. As such, discussing access needs in front of other colleagues is a nonconsensual sharing of personal information. Several interviewees also described being forced to disclose access needs to explain to coworkers why work wasn’t being done in the expected way. For example, Raegan Swanson, a second-generational white settler with an invisible physical disability and learning disability, recalled asking for help and being questioned by her coworkers about why she needed help with a task she had previously done:

They had seen me move boxes previously and they are like, well, why aren’t you helping today? And it’s like, well, today, today I cannot move. Like, I’m in an extreme amount of pain and having to go through it all with them and like the personal details of how pain works to try to justify my request to them.

Participant 2 similarly described multiple experiences at work where, “I’m literally in a position where I’m forced to disclose, or there’s going to be a question of why I’m not doing that aspect of my job.” Many interviewees expressed frustration, feeling like they needed to disclose personal medical information to colleagues in order to get their access needs met at work. For example, Joy was required by her employer’s human rights office, who managed accommodations, to repeatedly submit medical documentation to support her accommodation request. As she explained, “They made me submit so many documents. Just every time it was like and more documents. They’re like, *oh no, it’s so easy, just this document from your doctor.* So, I submitted that. And then it was another… that just went on so long, honestly months.” Eventually, Joy had to involve her union representative to get approved for an accommodation she described as “not even hard” to administer. Similarly, Participant 4, a white, cisgender woman who has non-epileptic seizures, described having to get detailed, third party documentation to validate what she felt was an observable disability experience. As she reflected: “This is just not great, right? I have to rely on my relationship with my boss, who, you know, can see that I am in a lot of pain essentially, trying to go through this process. The fact that I have to open up my whole medical history to people [HR staff] who are, like, essentially insurance brokers.” For these professionals, the need to disclose was compelled by organizational cultures where ability was presumed to be static or where colleagues were, in some cases, empowered to manage, question, and even push back on requests for access.

Even in situations where their requested accommodations were approved, some participants described having to repeatedly advocate for their implementation. For example, Participant 2 continuously struggled with coworkers in the company’s technology department over access to real-time captioning in virtual meetings: “I’ve said that, like, you know, I cannot caption myself. I’m hard of hearing. I do not know what more I can say.” Participant 2 shared how even after frequently raising their access needs with colleagues, “there’s still no move to accommodate it. There’s no move to add closed captions to our webinars or Zoom meetings or anything like that. There is no advocacy on behalf of my supervisors. Like, nothing changes.” Another participant, Participant 4, relied on an elevator to move around the building and retrieve items from the archive where they worked. They described how the elevator was often not fully functional (and never fully repaired), which meant they regularly needed assistance to operate it. As they explained: “most days I have to call somebody while I’m in the elevator to go up and down,” which meant that over the course of the 4 years they had been working in that archive, “everybody’s watched me struggle to do this stupid thing,” which required them to repeatedly ask for assistance from their colleagues. This experience of being forced to struggle with an inaccessible environment was also shared by Zachary, an Autistic, white, cis, heteroflexible man, who described how, even after clearly and carefully articulating his access needs during an interview process, accommodations were not provided: “There was a point in the interview where I’m just like, ‘I know that I’m not doing well, but I asked to know who I would be meeting with. I asked to know what the questions would be. I did not get those things. And so I am struggling.’” For Participant 4 and Zachary, the resistant culture around accommodations meant not only having to repeatedly disclose, but also having access needs repeatedly put on display in front of colleagues because their accommodations were not provided.

This first finding illustrates the multitude of ways that the people who we spoke to experienced forms of forced, repeated disclosure. Some described the invasive process of having co-workers share details without consent, while others spoke about having to continually ask for the accommodations they need and nonetheless have them denied or ignored. These experiences highlight ways that ableism is embedded in some professional workplaces through the lack of accommodations, the denial, neglecting, or “forgetting” of accessibility measures, as well as the interpersonal ways that these are enacted.

#### 4.1.2 Forced to conceal and “power through”

In a context where accommodations were challenging to secure and inconsistently implemented, several disabled archivists described feeling forced to conceal their access needs and “power through” by finding ways to complete their work without accommodation. These decisions were often motivated by the recognition that access was a scarce resource that needed to be strategically rationed and politically managed through relationships with colleagues. For example, Zakiya Collier, a Black, queer, chronically ill, and disabled cisgender woman living with systemic lupus erythematosus (SLE) and other autoimmune and long-term conditions, recalled weighing the limited sick days they had available when considering whether to go into work when their chronic pain was higher (Brilmyer et al., 2024, p. 123):

I tried to sometimes power through and like save my sick days. Cause I do not know if there’s like a extended sick period coming up. And so it’s like, it just feels very like I’m rationing my like… kind of doing a scale like they do at the doctor’s office for myself. Like, is it [my pain] a seven? Okay. You know, like if it’s seven and above, you should take sick time. If not, power through and just like be in pain to, to like avoid the stress of like figuring out what would happen next.

Another resource that interviewees perceived as scarce was their colleagues’ understanding and support around access. In particular, some interviewees expressed concerns about how their supervisor would respond to accommodation requests. For example, Chris Tanguay, a queer, white, gender-ambivalent woman with reoccurring depression, generalized anxiety disorder, and thoracic outlet syndrome, felt like they could *not* refuse to do tasks that caused them pain because of the anticipated response from their former supervisor. They told us, “I do not think it was necessarily the disability, but at the same time, I feel like if I said I cannot do that, she would’ve just responded, ‘I have no sympathy for that. You know, this is what you were hired to do.’” Jade Finlinson, a white paraplegic with spinal cord injury who uses a wheelchair for mobility, similarly worried that repeated requests around access might lead their supervisor to perceive them as “not having the skills to do it [their job].” (Brilmyer et al., 2024 p.130).

Interviewees’ deliberations over whether to try and “push through” without accommodations spotlight the power dynamics that emerge as supervisors can function as gatekeepers around access at work. Several participants considered how (in)secure they felt in their current position when making decisions about how to navigate access barriers in the workplace and whether to advocate for accommodation. For example, one archivist, Chris, described feeling like they could not say no to tasks that left them in pain for days afterwards because they were hoping to be promoted. As they elaborated: “I felt like I had been given my current position as a favor. … I did not feel like my boss liked me. … So I would kill myself trying to get [the top review] and, you know, trying to be the good worker and not make waves.” Chris had been able to secure an informal accommodation from their new boss that allowed them time off for therapy appointments. They had previously been unsuccessful in getting a formal accommodation approved through human resources “because the HR representative did not want to fill out the paperwork for it.” Although their boss approved their informal request to flex time, “I also worry like, well, this is not written on paper anywhere, so this could come back to bite me, especially since I’ve been active in unionization.” This concern that access arrangements at work were conditional on the goodwill of their supervisor was similarly echoed by Participant 2, whose sense of job precarity factored into their unwillingness to “fight” for their accommodations: “I’m not willing to completely fight for it until I’m in a really stable position where I know that that’s not gonna cause me to like, not have a contract renewed or like not get tenure or something.” They told us about how they had waited to disclose and advocate for accommodations until felt their job was secure: “I’d also passed probation at that point. So, I knew like it would be difficult to fire me if they wanted to for that.”

In summary, within some hostile and/or ableist workplaces or workplace cultures—where some participants described having to continually disclose their disabilities in order to get accommodations or where accommodations were routinely ignored or denied—some participants also described choosing to “power through” or deny their own needs. This response was described by some as rationing time off, choosing not to repeatedly ask for accommodations, or not feeling as if they could say no to tasks in order to keep one’s job in a precarious landscape.

#### 4.1.3 Forced to manage the emotions of others

Adjacent to the theme of relational power dynamics shaping how disabled archivists advocated for access was how interviewees described needing to manage the emotions of others while negotiating access. Interviewees identified worry as the primary managing emotion that emerged as they advocated for access at work. Several worried that colleagues would perceive their need for access as ‘too much.’ For example, when discussing how it felt to advocate for herself, Michelle stated:

My thing is, I’m always worried that I’m overstepping the bounds of the kindness that they are giving me. Which is an incorrect way to feel because the things I am asking for are not so unique or costly or difficult or time consuming, that I should feel like it’s a burden. But it still feels that way until I get the reaction from the person I’m asking.

Even Joy, who served as a director, had not brought up her access needs even when her employer was “asking us for input” because “I’m just worried about it.” This worry was common for participants and had several dimensions to it, which included concern around drawing negative attention and being perceived in a negative light. For example, Participant 5, a disabled, gay Black man with chronic back pain who often depends on muscle relaxers to perform daily tasks, recalled hesitating to use seating accommodations at an archival conference, explaining: “I do not think people would see me as somebody who needs to be sitting in the accessible seating area, you know? … Maybe I ‘should not be so caught up in their perceptions,’ but I think certainly at [a professional conference], when you go there, it’s paid for by your employer, you, you have to certainly be a certain way, right?” Chris similarly reflected on the physical lifting that was included in their job description and how they did not want to “draw attention to the fact that I struggle with that sometimes.”

Another dimension of interviewees’ worry was that they had low expectations about colleagues’ capacity to understand and accept access needs, particularly ongoing and changing needs. For example, Zakiya worried about their colleagues’ capacity to understand the episodic nature of their disability, and felt burdened by the pressure to predict their access needs accurately: “having to always think about like, ‘Am I going to have to explain this to somebody? And will it make sense?’ Because it does not make sense to me all the time.” Similarly, Participant 4 spoke about how the experience of “asking people again and again and again and again” about access was emotionally stressful and meant “I am used to being extremely patient because I’m forced to be all the time.” Over time, they described how the experience of predicting and catering to colleagues’ anticipated responses “feels like I’m doing this like kind of like mothering, or emotional labor where I’m constantly questioning what does this person respond to? What approach should I take?” Finally, Raegan, who has invisible learning and physical disabilities, spoke about having to get used to colleagues’ negative attitudes towards her access needs, “dealing with people being extremely rude or doubting like what I have to say,” and figuring out “how to not take that too personally while like I’m trying to either do my work or, you know, just live my life” (Brilmyer et al., 2024, p. 124).

On the rare occasion when interviewees received support from colleagues, it was notable that they framed this access labor, even if it was partial or inadequate, as nice or as acts of kindness. For example, Participant 4 shared how a colleague’s unsolicited offer of help surprised them because it meant that person had noticed the episodic challenges associated with her disability. As she reflected: “That’s really nice that they actually understand on this level that it’s a stress that I am dealing with constantly, which I did not really expect. … It’s hard to tell what people think, but it feels like there’s a lack of recognition.” Still, she elaborated that even this provision of access produced complex and conflicting feelings:

The genuine gratitude I feel, regardless of whether necessary, is complicated further by THEIR feeling of ‘being nice’ or the social exchange that is expected out of this. This feels cynical—though is still a reality—on an interpersonal level, but on a social one it can easily put me in a kind of debt—i.e., perception that I cannot be deserving of accommodation or recognition and be anything less than grateful at the same time.

Put another way, she was mindful that her colleague’s feeling of ‘being kind’ posed its own emotional demand that forced certain kinds of responses, such as performing appreciation or gratitude. Across interviews, only one participant, Jade had recalled feeling supported by their supervisors, as they explained: “That made a big difference, just to feel that even if there were going to be problems–and we all acknowledged that there would be problems and that I would need help, and that I would have to ask for help for certain things–and that was okay. And so I felt very supported.”

In summary, these words highlight the ways the disabled archival workers that we spoke to experienced and managed emotions as they navigated accommodation processes. Some participants described worrying about if their accommodations requests would be seen as “too much,” while others chose not to disclose so that they would not encounter such attitudes, expecting that colleagues would not understand or be accommodating. Finally, interviewees described gratitude as a particularly complex emotion because it was sometimes performed out of obligation or anticipated discrimination but could also be genuinely felt when access and support were provided.

### 4.2 Affective impacts and responses

#### 4.2.1 Emotional responses: stress, fatigue, and hurt

Archivists described hurt and stress as the primary emotional toll of battling consistently inadequate and complex accommodation processes that slowed or inhibited access in their workplace. Archivists like Raegan spoke about the toll of “navigating the bureaucracy of filling out forms for disability services and stuff like that with the government” and how it factored into her “general exhaustion” when she is at work. Michelle shared how ongoing workplace stressors, including inaccessible spaces but also challenging and discriminatory organizational dynamics, took a physical and emotional toll: “it was super unhealthy. My blood pressure was too high. My stress levels were too high. My weight was too high. Everything was too high.” Participant 4, who had experienced a seizure related to stress at the office, described “getting really upset” when her department not only consistently failed to meet her access needs but also, through this, added to her stress. As she explained “I kept being like, ‘This thing is not working, this thing is not working’ … There should be better ways that we can deal with these conflicts or long-ignored stresses or interpersonal things that kind of led me to this point.”

For several archivists, the emotional labor of navigating access in the workplace was holding feelings of being hurt after humiliating interactions with colleagues. For example, Raegan described encountering ableist assumptions from supervisors after requesting extra time to review written work (Brilmyer et al., 2024, p. 117):

I’ve had employers … who go, ‘Oh, do you even know to like write? Can you write like words?’ And I’m like, ‘I have a fucking master’s degree. I need you to think about what you say before it comes out of your mouth.’ … And so that in a professional workplace has been extremely unsettling.

This type of condescending attitude was also part of Michelle’s experience at work, who had been chastised after a challenging incident “where there were some chaos going on and I was trying to listen to the phone call while talking to someone–which is not a thing I can do.” Their coworker had aggressively intervened and “grabbed the phone out of my hand” and later “informed me that I just need to ‘figure out my disability, cause that was unacceptable.’” Michelle recalled feeling really hurt by that interaction: “I went home crying that day and it was not, not cool” (Brilmyer et al., 2024, p. 116).

Interviewees reflected that working in inaccessible spaces and dealing with hostile workplace cultures contributed to their stress. This hostility was also harmful, causing physical and emotional pain that only compounded the existing inaccessibility of the office.

#### 4.2.2 Pushed to the limit: refusal

Several archivists described getting to a place where they decided not to continue engaging in accommodation processes at work and these decisions were largely framed as refusing to continue engaging in the emotional labor produced by an ableist culture. As Participant 4 succinctly expressed while describing their frustration at how a simple access request had morphed into multiple medical appointments just to get supporting documentation: “I was just like, fuck this. I’m not subjecting myself to this bullshit for them to literally give me a key to a door that I can covertly open anyway, but of course there are different types of risks.” Participant 2 similarly explained how, “once it passes a certain level or a certain threshold of labor for me, I just give up. Which is not the greatest way to deal with things, but I definitely do it. I just get too stressed out and I just—I do not have the patience or energy to deal with this anymore.” These expressions of running out of patience and interest in subjecting themselves to exhausting administrative labor hint at the constrained agency of disabled archival workers in transforming or avoiding the emotional labor of accommodation processes; their agency was their ability to refuse to participate entirely as a strategy for self-preservation.

This strategy of refusal was similarly evident in Michelle’s decision to avoid professional spaces where she anticipated her access needs would not be met, as she shared: “there were a couple of events I just would not go to because I’m like, I’m not going to be able to hear anything. All I’m going to do is, you know, feel uncomfortable and be bored.” Over time, the cumulative effect of these experiences was that Michelle had considered more broadly leaving archival institutions, “just bailing on the field altogether,” in anticipation that the inaccessible culture would not change, even though “so much of who I am is an archivist.” Both Michelle and Zakiya shared that they knew of disabled colleagues in archival institutions that had left the field because of a lack of access. Zakiya explained how their colleague’s decision to leave after being denied a requested accommodation to attend therapy,

…told me a lot about, you know, where I was working and like what their values are. And I’ve been able to like, communicate that to other people, like this is not a completely safe space. It’s accessible legally, but culturally not so much. I’m just like, why would you want someone to not be mentally well at work?

Their critique of the organization’s values echoed a sentiment that came through across many interviews: that the difficulty accessing accommodations reflected a deep-seated culture of ableism in the workplace that prioritized abstracted ideals of ability over employees’ wellness, safety, and basic needs.

In response to the pain, hurt, and exhaustion they experienced through accommodations processes (or lack thereof), several participants chose to stop participating in these processes. These acts of refusal included declining to provide additional medical documentation to support an accommodation request, running out of patience and “giving up” on negotiating accommodations,^4^ and leaving a workplace and even the archival field altogether. These refusals functioned as forms of self-preservation in the face of processes that were burdensome, unproductive, and ultimately hostile to disabled workers.

#### 4.2.3 Finding confidence by contributing to a culture of access

The lack of effective accommodation processes at most archival institutions represented in this study meant that disabled archivists regularly felt forced or compelled to repeatedly disclose disability information with colleagues and also to “power through” without accommodation. Archivists described the process of navigating ableism at work as a battle, something they had to fight for with their supervisors and colleagues. By contrast, when participants had opportunities to advocate for access to benefit other disabled archivists or to build community with and for other disabled archivists, they described those experiences as creating a sense of ease and satisfaction. For example, Raegan described advocating for an update to her institution’s human resources policies to make the processes for requesting accommodations clearer. Initially, “there wasn’t anything about accommodations in the HR policy. So like, that was something that I went in and made sure was added so that when folks come in, they know that this is the kind of space where they can talk about it or ask for things.” When asked how it felt to update the policy, she said: “it was very satisfying to me to be able to put that in.”

Similarly, Joy shared how it felt “easier for me to advocate for others,” knowing “I’m not just fighting for myself for this thing I can talk myself out of not really needing, but somebody else might use this physical thing too.” She explained that knowing “someday, somebody will benefit from this even if I do not,” which made it easier to process the harms and hurt emotions that came up in fighting for access. Joy also described how interacting with other disabled co-workers or students and sharing stories and challenges around accommodations had helped her:

Students talking about what their experience was really amazing. I was like, ‘oh crap, this is not even difficult.’ It was so easy to see that like, wait, either there’s something wrong with me that I feel that this is very, very reasonable, or there’s something wrong with them [the employer].

Although, on an individual level, Joy would often respond to having her access needs dismissed or challenged by minimizing and doubting the importance of those needs, observing others advocating for what she needed empowered them to continue advocating for change at work. This was similar to Chris’s experience, where finding community with other disabled people had empowered them to continue pushing for what they needed at work. As they explained, “It’s good because I learn more about the things that I can ask for. … Being in a community where people actually actively talk about self-advocacy, I feel like it kind of legitimizes a lot of my thoughts.”

This second cluster of findings shows the variety of responses to the accommodations process in archives. Participants described a range of affective responses to accommodations processes: from stress and hurt to fatigue and exhaustion. Being pushed to their limits, participants also described being fed up or refusing to participate in harmful processes. Yet, they also described feeling empowered when contributing to a broader culture of access in their workplace, one where they could depend on others to support their access needs, share access labor by advocating for others, and collaboratively build accessible workplaces.

## 5 Discussion

This research outlines the complexity of disabled archival workers’ lived experiences—the ways they navigate accommodations and the affective impacts of employment, workplace policies, institutional culture, and professional norms. Across conversations, the archivists we spoke to described negotiating ineffective systems for work accommodations and having to weigh complex considerations around how and when to formally request an accommodation or informally raise access needs with colleagues. For participants, negotiating access was highly political: for example, several archivists spoke about waiting to raise access needs until they felt more secure in their position out of fear of retaliation. Even when accommodations were approved, interviewees often had to proactively and continuously advocate for those accommodations to be implemented. Their stories align with previous research documenting the repetitive administrative and relational labor that is required to secure and maintain disability-related accommodations and benefits (Damiani and Harbour, 2015; Konrad, 2021; Price, 2021; Titchkosky, 2011).

### 5.1 How organizations (En)force emotional labor within accommodation processes

Hochschild (1983) frames emotional labor as how organizations use and demand emotional expression from their workers. Our research considers how organizational accommodation processes and norms around workplace access demanded certain types of emotional expression and management. In doing so, we build on scholars’ theorizing and documentation of access labor, what Fink (2020) has defined as “the work and effort that goes into making things accessible.” Interviewees referenced several types of access labor that they felt compelled to engage in as they negotiated access at work, such as being forced to repeatedly disclose their disability and access needs, being compelled to conceal access needs and “power through” without accommodation, and being forced to manage the emotions of others.

These experiences of access labor, first, exemplify what Mingus’ (2017) has conceptualized as forced intimacy, as in the “common, daily experience of disabled people being expected to share personal parts of ourselves to survive in an ableist world” (para. 1). Forced intimacy emerges out of the relationality of access, and the how access often becomes dependent on the way non-disabled people feel towards the person requesting accommodation (Mingus, 2017). As a result, disabled people are often forced to be vulnerable by exchanging personal information for basic access in formal spaces, like work and school, as well as less formal spaces, like being at the grocery store or on public transit. While Mingus describes the intimacy of access as having the potential to be “magnificent,” “powerful,” and “transformative” when mutually embraced through a politics of love and solidarity, in the context of an ableist world, this intimacy is often experienced as a loss of consent as well as a source of frustration and harm. In this “caged reality,” Mingus (2017) explains how disabled people are expected to manage the emotions of others to survive, for example by being friendly to strangers, responding to harmful actions with patience and forgiveness, or performing gratitude for “whatever crumbs [of access] are thrown our way.”

The findings in this study demonstrate how forced intimacy was a normalized experience for archivists with disabilities in professional spaces. Archivists relied not only on administrators and supervisors who were involved in formal accommodation processes but also colleagues who were involved in the day-to-day facilitation of access; for example, turning on captions for Zoom meetings or providing assistance in operating an elevator to navigate through the archives. As a result, several interviewees’ were not only compelled to discuss disability information and access needs but also were forced to struggle with access barriers in real-time in front of colleagues and co-workers, as Raegan described, “having to go through it all with them and like the personal details of how pain works to try to justify my request to them.” As Mingus (2017) explains, forced intimacy “is a cornerstone of how ableism functions in an abled bodied supremacist world. Disabled people are expected to ‘strip down’ and ‘show all our cards’ metaphorically in order to get the basic access we need” (para. 3). Beyond the intimacy inherent within having to disclose personal information to coworkers, interviewees also described worrying about how colleagues and supervisors would react to access requests and needing to negotiate access patiently while expressing gratitude for minimal accommodation.

The second key contribution of this study is around the emotional management and labor that is embedded within formal and informal navigations of access at work. This study not only aligns with Mingus’ (2017) depiction of emotional labor as part of forced intimacy but also extends previous research with disabled people that has documented emotional labor as a central dimension of negotiating access and navigating ableism day-to-day (Konrad, 2021; Price, 2021). These scholars highlight how despite access being framed as largely procedural or logistical, it is inevitably dynamic and relational work that takes an emotional toll (Price, 2024; Titchkosky, 2011; Valentine, 2020). For example, in their work with people who are blind and visually impaired, Konrad (2021) highlights how the rhetorical demands of negotiating access–such as teaching others about access, performing a palatable disabled self, and dealing with people’s reactions to disability–-produced fatigue. Specifically, they coined the phrase “access fatigue” to name the everyday labor of “constantly needing to help others participate in access” but also the ongoing demand to care for others’ emotional experience as part of this helping process. Many of the archivists in this study similarly highlighted how the management of emotions was a central dimension of negotiating access at work. This management included holding and experiencing internal emotional states like stress, hurt, and fatigue but also strategically deploying certain affects, such as gratitude and patience, to manage the emotions of colleagues, as Michelle told us, “I’m always worried that I’m overstepping the bounds of the kindness that they are giving me.” This research thus also adds to emotional labor, empathy, and relationality of archival work (e.g., Regehr et al., 2023; Nathan et al., 2015, Laurent and Wright, 2023, Caswell and Cifor, 2021) and the emotional management that is sometimes part of (or expected for) archival work.

### 5.2 Surface acting, emotional costs, and emotional gifts as central dimensions of access labor

There are several concepts from studies on emotional labor that are useful in theorizing the emotional experiences of disabled archival workers with access and accommodation in the workplace. This section will explore three: surface acting (Hochschild, 1983), emotional gifts (Clark, 2004), and emotional costs (Price, 2024). Participants described engaging in a form of surface acting: the transmutation of negative feelings like frustration into affects like patience and gratitude to manage the emotions of their colleagues. In this way, disabled archivists regulated their affects to ensure the cooperation and compliance of others in workplace accommodations. This emotional management was consistently perceived by disabled archival workers as an implicit, “affective requirement” of negotiating access to work (Wharton, 2009, p. 158). Price (2024) describes this as “impression-management work” and explains how masking inner worry, hurt, and stress with performances of positive or neutral emotions can, itself, exacerbate the emotional pain of negotiating access (p. 117).

This management of internal and external emotional experiences affirms an understanding of emotion as “flow[ing] between and among people” in ways that are “patterned rather than random” and often reflect relative positions of power (Clark, 2004, p. 403). Clark (2004) notes that the logics of “feeling rules” are gendered, and this study demonstrates that emotional experiences are also patterned based on disability status. For the disabled archivists in this study, their emotional labor was directed towards coworkers and supervisors, whose participation was necessary for accommodations to be implemented. This echoes previous research on disabled women leaders in the workplace, which identified surface acting as a relationship management strategy that women with disabilities used with coworkers, regardless of their seniority (Boucher, 2017). Surface acting within the accommodation process reflects the relational and precarious nature of disabled workers, who use affective strategies to prove and perform deservingness within ableist workplace cultures that treat access as a burden and practice of charity (Gerrard, 2019). Interviewees described making decisions around access based on the anticipated reactions of colleagues and supervisors, in particular, interviewees worried about their needs being perceived negatively, as ‘too much’ or overstepping. Anticipating being perceived negatively, interviewees were compelled to conceal their access needs and “power through” without accommodation to avoid negative emotions, dismissive reactions, and harmful judgments, exemplifying what Ahmed (2012) describes as practices of “institutional passing” or “going along with” (p. 157). Clark (2004) frames positive emotions like gratitude, respect, and deference as gifts, “emotions that one social actor expresses or displays (verbally or nonverbally) to another that have value because they are scarce—that is, they are not giving indiscriminately or limitlessly—and because they create positive emotions in the other” (p. 404). On the surface, this framing is useful in theorizing why disabled archivists might offer patience and gratitude to a coworker or employer to build up “socioemotional credits” that can be effectively traded for cooperation and assistance. However, Clark’s use of gift feels inadequate in conveying the weight of feeling forced to exchange of positive emotions for basic material needs, such as safe and equal access to the workplace. For example, Clark describes how “a social actor who fails to receive expected emotional gifts might feel slighted and in turn withhold his or her own emotional gifts,” but what is at risk of being withheld in interactions around access is not only reciprocal emotional gifts, but employees’ safety, dignity, and ability to work.

Furthermore, the internal emotional experiences of disabled archivists as they navigated access at work came at a significant cost to participants’ well-being. Price (2024) borrows from understandings of personal cost and emotional labor in describing emotional costs as the negative emotions, such as sadness and anger, that are experienced in relation to navigating access. These emotional costs were evident in the experiences of disabled archivists in this study as well. Beyond feelings of frustration, hurt, and sadness, participants also described the weight of internalizing the ableist logics through which they were perceived (and devalued) in order to strategically navigate workplaces defined by these logics. These experiences reflect what Titchkosky (2011) identifies in Audre Lorde’s writings on anger, how she was “forced to incorporate a response to others’ destructive recognition of her into her self-understanding” (p. 146–7). And while participants negotiated around and through ableist logics, several described hitting the limit of their capacity to tolerate the emotional management demanded by accommodation processes—opting out of a formal accommodation process entirely, declining to participate in work-related events, and even considering leaving the field.

Yet, while refusal or opting out of traditional accommodation processes can function as a strategic move for survival and self-preservation for disabled people (Damiani and Harbour, 2015; Emens, 2021; Karpicz, 2020), the emotional labor demanded of the disabled archivists we talked to functioned as what Emens (2021) describes as “a hassle cost,” a way of rationing access by making it impractical and challenging to request and secure accommodations (p. 2348). The emotional labor required to survive and secure basic access in archival institutions was demanded in both formal accommodation processes and in day-to-day interactions with colleagues and supervisors, effectively serving as a hassle cost that preserved a status quo that privileged and legitimized the labor of nondisabled professionals. These demands—for patience, vulnerability, worry, and gratitude—formed a unique affective landscape for disabled archival workers that had to be continuously performed and managed as part of negotiating access, affirming the harmful and uneven impacts of surface acting for marginalized workers (e.g., Glomb and Tews, 2004; Ozcelik, 2013).

### 5.3 Collective approaches to access shift the affects of access labor

It is also significant that the emotions disabled archival workers experienced around collective approaches to access—ease, empowerment, and confidence—differed from those they experienced and navigated under traditional accommodation processes, which were either negative or paternalistic. This contrast reinforces that approaches to access are encoded with possible and permissible emotions. Collective approaches to access, because they are rooted in solidarity and embrace disability culture, produced positive affects that were neither performative nor superficial. Contributing to a culture of access within the workplace affirms access as both a collective responsibility and a shared asset (Hubrig and Osorio, 2020; Long and Stabler, 2022; Fritsch, 2024).

On the contrary, traditional approaches to individual accommodation reinscribe access as charity. This was evident in Participant 4’s awareness of how providing access created a feeling of “being nice” that functioned as a demand for gratitude. As she explained, a social and emotional debt was produced when access was provided, namely that “I cannot be deserving of accommodation or recognition and be anything less than grateful at the same time.” Thus, the findings of this study contribute to an understanding of how organizational approaches to access structure, as in shape and constrain, affect.

### 5.4 Theorizing “emotional expense” and indebtedness within archival organizations

Importantly, this paper outlines an *indebtedness* that emerges through accommodations processes because of and in response to inaccessibility at work. Interviewees incurred an external indebtedness—to colleagues, human resources, other people involved in the bureaucracy of access —‘paying’ with gratitude, giving vulnerability, being patient, and nice, to be deserving of accommodations. This external indebtedness emerged in the ways participants worried about exhausting their colleagues’ willingness to negotiate access, reinforcing the idea of access as a form of benevolence that is conditional on not “overstepping the bounds of the kindness that they are giving to me” as Michelle explained. Yet, this also included an internal indebtedness—borrowing against your patience, energy to survive, and work—which was reflected in interviewees narratives about running out of these internal resources. This internal indebtedness expands upon Price’s (2024) conceptualization of emotional cost as involving both external or “human-to-human” personal costs as well as internal negative emotions that were not tied to feeling indebted to another person (p. 114). This study expands this concept of emotional cost by demonstrating how indebtedness, not just negative emotions, is experienced internally as part of negotiating access at work. Together, these external and internal emotional debts underscore the frictions produced through inaccessible workplaces and bureaucratic and demanding accommodation processes, as well as the way disabled people incur emotional tolls as they navigate constrained and uneven relationships. To be in such emotional debt shapes an affective landscape, as Participant 4 articulates, navigating the ableist power dynamics around access, “can easily put me in a kind of debt—i.e. perception that I cannot be deserving of accommodation or recognition and be anything less than grateful at the same time.”

Building on Brilmyer’s (2022) articulation of the “emotional expense” of archival inaccessibility for disabled archival users, we draw attention to *the affective debt of archival access*: the complexity of inaccessibility, where such expenses—because of the requirements to manage internal emotional costs on top of system demands, bureaucratic processes, and interpersonal interactions with colleagues—create many types of indebtedness, many (if not all) of which are required to keep ones job. We outline the debt of such affective demands—emerging across different archives and therefore showing the prevalence in the field—to illustrate how many of the interviewees developed a deep awareness around having to share details about their disabilities in order to gain accommodations, deal with harmful processes and people, and manage the futility and incurring cost of it all. This term aligns and builds with Cuellar et al.’s (2023) conceptualization of “archival debt,” which they describe as the “problematic legacy issues” that have accumulated over time as institutions take shortcuts and make compromises in archival practice, such as “harmful or inadequate description, performative or competitive collecting, languishing backlogs, failure to recognize staff potential, shortsighted fund management, neglected constituencies, a lack of documentation, and poor project management” (p. 1). The findings of this study expand on this concept of archival debt by documenting how delays and shortcutting accommodations processes preserves the inaccessibility of archives. For example, delayed and underresourced accommodations shape who can work, who gets promoted, whose contracts do not get renewed, and therefore who are the stewards of archival materials and access to histories. The concept of *affective debts of archival access* also captures how indebtedness preserves the power dynamics that contribute to disparate emotional experiences; disabled workers continue to be put in the position of requesting access at an individual-level and coworkers and supervisors continue to be empowered to invalidate, delay, and/or deny access needs.

The concept of *the affective debt of archival access* affirms how phenomena like forced intimacy (Mingus, 2017), institutional passing (Ahmed, 2012), surface acting (Hochschild, 1983), and emotional hassle costs (Emens, 2021) emerge as disabled archival workers maneuvered accommodation processes and the emotional labor that was exacted through these processes. Participants described feeling forced to experience or unable to avoid this emotional labor, whether it was experiencing the vulnerability of repeatedly disclosing personal information or managing the emotions of colleagues as they provided, contested, and/or denied access. Though the affective demands of accommodation processes felt unavoidable within traditional accommodation processes, disabled archival workers described how collective approaches to access transformed the affective experience of access towards ease and empowerment. Their narratives affirm that different affects are facilitated through workplace structures and cultures. Within archival institutions, the affective demands on disabled archival workers were deeply shaped by structures and cultures that preserved abledness as the norm.

Building on the literature that highlights the affective aspects of archival work (e.g., Arroyo-Ramírez et al., 2021; Caswell and Cifor, 2016; Guerrero, 2022; Laurent and Wright, 2023), we highlight new facets of archival labor related to accommodations and access, how they are often invisibilized (Lapp, 2019), and how this shapes how and if archival work is done. By drawing attention to how the very people who facilitate access to historical documents are also navigating their own access, this research, then, connects the workplace conditions for disabled archival workers with the experiences of users (e.g., Brilmyer, 2021, 2022, Brilmyer et al., 2024; Duff et al., 2019).

## 6 Conclusion

The findings from this study affirm how, as Ahmed (2006) theorizes, “spaces acquire the ‘skin’ of the bodies that inhabit them,” meaning that organizational norms and cultures are shaped by the people who have historically occupied and preserved their power within and through institutions (p. 132). And though individual accommodations may temporarily modify the ‘skin’ of an organization, they rarely alter its culture such that disabled people truly feel a sense of welcome and belonging (Piepzna-Samarasinha, 2018). In sharing their experiences with access at work, interviewees documented how archival institutions of all kinds—whether community-based archives, university archives, large or small collections—had embedded organizational norms that presumed and privileged abledness. Within these cultures, archivists experienced a broad range of access barriers: from a lack of information about how to request accommodation as a job candidate to having colleagues repeatedly forget to turn on the captions, an approved accommodation, in virtual team meetings. Abledness was enforced as the norm and made compulsory through this difficulty (McRuer, 2018). Disabled archivists found themselves continuously bumping into entrenched understandings of “that which is expected” of archival workers and archival work, which did not include either the labor of providing or the experience of receiving accommodation (Garland-Thomson, 2011, p. 593). Interviewees largely had low expectations for the quality of access they would receive and navigated with an awareness that every request for access could become a protracted struggle with supervisors, colleagues, and administrators.

*The affective debt of archival access* draws attention to how these struggles to negotiate basic access at work revealed “sedimented patterns of relating and belonging” within these institutions that forced disabled archivists to regularly disclose personal information and advocate for their access needs to survive in the workplace (Valentine, 2020, p. 77). Negotiating access in this context resulted in a complex affective landscape for disabled archival workers. In particular, this study contributes to understandings around the emotional cost of access by documenting the internal debts that accrue as disabled archival workers navigate access at work. Participants’ narratives highlight how these affective politics are normalized for disabled workers in archival spaces, where workers feel obligated to make their hurt and anger small and then transmute it into patience and gratitude in order to get the basic access they rightfully deserve—producing internal and external indebtedness, costs that aaccumulate across the archival profession.

## Data Availability

The original contributions presented in the study are included in the article/supplementary material, further inquiries can be directed to the corresponding authors.

## Footnotes

^1^For example, nationally, the Accessible Canada Act, although not universally applied across Canada, aims to create barrier free access to the public or the public sector, and the Americans with Disabilities Act requires that public spaces have “a path of travel — safe harbor” to public spaces including government buildings, educational settings, and public transportation.

^2^Many archival workers complete advanced degrees, frequently in library sciences or history, prior to entering professional archivist roles.

^3^Sloan et al. (2019) note “the extent to which archivists are expected to remain neutral and objective, and to maintain a certain distance between themselves and the records with which they work” (p. 15).

^4^Earlier in the findings, we identified “powering through” as one way that disabled archivists navigated (a lack of) access in their workplace. While the outcomes of “powering through” and “giving up” were similar: disabled archivists found ways to work without formal accommodations. However, participants described the purpose of these practices differently: “powering through” was a strategy for getting work done when access was scarce, “giving up” was a move away from the accommodation process itself. The latter took on a self-protective quality, as in refusing to continue engaging in harmful negotiations and the pressure to produce more and more documentation.
